# *In vitro* biocompatibility evaluation of functional electrically stimulating microelectrodes on primary glia

**DOI:** 10.3389/fbioe.2024.1351087

**Published:** 2024-01-19

**Authors:** Christopher T. Tsui, Soroush Mirkiani, David A. Roszko, Matthew A. Churchward, Vivian K. Mushahwar, Kathryn G. Todd

**Affiliations:** ^1^ Department of Biomedical Engineering, University of Alberta, Edmonton, AB, Canada; ^2^ Neurochemical Research Unit, Department of Psychiatry, University of Alberta, Edmonton, AB, Canada; ^3^ Neuroscience and Mental Health Institute (NMHI), University of Alberta, Edmonton, AB, Canada; ^4^ Sensory Motor Adaptive Rehabilitation Technology (SMART) Network, University of Alberta, Edmonton, AB, Canada; ^5^ Department of Biological and Environmental Sciences, Concordia University of Edmonton, Edmonton, AB, Canada; ^6^ Division of Physical Medicine and Rehabilitation, Department of Medicine, University of Alberta, Edmonton, AB, Canada

**Keywords:** neuroinflammation, microglia, astrocyte, electrical stimulation, microelectrode, biocompatibility

## Abstract

Neural interfacing devices interact with the central nervous system to alleviate functional deficits arising from disease or injury. This often entails the use of invasive microelectrode implants that elicit inflammatory responses from glial cells and leads to loss of device function. Previous work focused on improving implant biocompatibility by modifying electrode composition; here, we investigated the direct effects of electrical stimulation on glial cells at the electrode interface. A high-throughput *in vitro* system that assesses primary glial cell response to biphasic stimulation waveforms at 0 mA, 0.15 mA, and 1.5 mA was developed and optimized. Primary mixed glial cell cultures were generated from heterozygous CX3CR-1^+/EGFP^ mice, electrically stimulated for 4 h/day over 3 days using 75 μm platinum-iridium microelectrodes, and biomarker immunofluorescence was measured. Electrodes were then imaged on a scanning electron microscope to assess sustained electrode damage. Fluorescence and electron microscopy analyses suggest varying degrees of localized responses for each biomarker assayed (Hoescht, EGFP, GFAP, and IL-1β), a result that expands on comparable *in vivo* models. This system allows for the comparison of a breadth of electrical stimulation parameters, and opens another avenue through which neural interfacing device developers can improve biocompatibility and longevity of electrodes in tissue.

## 1 Introduction

Neural interfacing devices interact with the central nervous system with the goal of improving a functional deficit from conditions including Parkinson’s disease and spinal cord injury (Volkmann et al., 2006; Groiss et al., 2009; Lavrov et al., 2015; Pikov et al., 2020). Devices used in interventions such as deep brain stimulation (DBS) and intraspinal microstimulation (ISMS) make use of thin invasive implants sometimes no thicker than a human hair, that are inserted into nervous tissue to physically make contact with and interact with cells (Eick, 2009; Sunshine et al., 2013; Arcot Desai et al., 2014; Herrington et al., 2016; Bamford et al., 2017).

Although such an approach is advantageous in that it allows for direct, acute and effective stimulation of neuronal networks (Guevremont et al., 2006; Bamford and Mushahwar, 2011), insertion of material into tissue inevitably elicits a foreign body response. In the case of the central nervous system, the foreign body response is characterized by microglia and astrocytes cordoning off the implant site from the surrounding tissue through the creation of a fibrous glial scar (Bérces et al., 2016; Wellman and Kozai, 2017; Keogh, 2020). The glial scar is problematic in that it prevents nearby neurons from accessing the implant for recording or stimulation purposes. Glia-driven inflammation persists in the tissue over several weeks to months which can lead to device failure and potential revision surgeries.

Over the past decade, there has been an increased effort to improve upon the biocompatibility of electrodes in the context of neural interfacing (Luan et al., 2017; Redolfi Riva and Micera, 2021; Frenzel et al., 2022). The main focus of such efforts has been on improving the material properties of the electrodes such as conductivity and mechanical stiffness (Dauzon et al., 2019; Shi et al., 2020). Other reports document the conjugation of biomolecules onto the surfaces of the electrodes to mask its foreign signature (Eles et al., 2017; Sridar et al., 2017). Antifouling compounds (e.g., zwitterionic polymers, polyethylene glycol) have also been reported in the literature to attenuate acute inflammatory responses elicited against invasive implants (Golabchi et al., 2019; Yang et al., 2020; Gori et al., 2021; Cho et al., 2023; Wu et al., 2023).

Although there have been reports on the effects of direct field electrical stimulation on glial cell lines *in vitro* (Kearns and Thompson, 2015; Pelletier et al., 2015; Vallejo et al., 2019; Lennikov et al., 2022); there is a paucity of reports documenting the effects of electrical stimulation on primary glial cells and associated cellular responses at the electrode interface. Evidence previously published by our group suggests that any responses from glia elicited by electrically stimulating electrodes develop acutely and are localized near the device interface (Bamford et al., 2010).

Here, we present a high-throughput and rapid means of assessing glial cell response to microelectrode implants *in vitro* via a hybrid cell biology and engineering approach. We recapitulated cellular responses observed previously *in vivo*—specifically, localized responses observed in tissue in proximity to the electrode in a longitudinal rat study by Bamford et al. (2010). In addition to assessing cellular responses to the presence of the electrode, we also determined cellular responses to different amplitudes of electrical stimulation. Extent of glial cell inflammation and damage was determined by immunofluorescence microscopy of specific biomarkers at and around the electrode. Furthermore, we assessed the extent of damage to the electrode itself through scanning electron microscopy and energy-dispersive x-ray spectroscopy.

## 2 Materials and methods

### 2.1 Materials

Dulbecco’s Modified Eagle Medium: Nutrient Mixture F12 (DMEM F12), Hank’s Balanced Salt Solution (HBSS), fetal bovine serum (FBS), penicillin streptomycin (PS), 0.25% trypsin-ethylenediaminetetraacetic acid (Trypsin-EDTA), and Equine Serum (ES) were purchased from Gibco (Life Technologies, Burlington, ON, Canada). Poly-L-lysine hydrobromide (PLL) was purchased from Sigma Aldrich (St. Louis, MO, United States). Polystyrene 12-well cell culture plates were purchased from Greiner Bio-One (Frickenhausen, Germany). Cell culture flasks (75 cm^2^) were purchased from Corning (Corning, NY, United States). Sylgard 184 polydimethyl siloxane (PDMS) kit was purchased from Dow Chemical (Midland, MI, United States).

Rabbit anti-IL-1β (Invitrogen, Burlington, ON, Canada) and chicken anti-GFAP (Abcam, Toronto, ON, Canada) primary antibodies were used. Donkey anti-rabbit Alexa Fluor 647 (Invitrogen) and goat anti-chicken Alexa Fluor 546 (Invitrogen) secondary antibodies were used. Hoescht 33,342, a nuclear stain, was purchased from Molecular Probes (Life Technologies, Burlington, ON, Canada).

Microwires (75 µm in diameter, Pt-Ir 80%/20% insulated with polyimide) for microelectrode fabrication were purchased from California Fine Wire (Grover Beach, CA, United States). Teflon-insulated, 9-strand stainless steel wires (Cooner AS632) were purchased from Cooner Wire Company (Chatsworth, CA, United States).

### 2.2 Cell culture preparation

Animal protocols were approved by the Animal Care and Use Committee at the University of Alberta and conducted in accordance with the guidelines of the Canadian Council for Animal Care. Mixed glial cell cultures were generated from the brain tissue of postnatal Day 2 C57BL/6J CX3CR-1^+/EGFP^ heterozygous transgenic mice (Jung et al., 2000). The mice were decapitated and their brains removed using surgical scissors and a metal spatula. Following dissection of the meninges using forceps, the remaining brain tissue was dissociated in 0.25% Trypsin-EDTA at 37°C for 25 min. The Trypsin mixture was then centrifuged twice at 500 g for 2 min and triturated in cell culture media (DMEM F12/10% FBS/1% PS) to further dissociate brain tissue and deactivate residual Trypsin-EDTA. The resulting cell suspension was placed in 12-well plates coated with PLL (2 μg/mL). Cells were incubated for 2 weeks at 37°C and 5% CO_2_, with cell culture media changed twice weekly.

At 2 weeks, mixed glial cells were washed with DMEM F12 and then lifted off from the 12-well plates with a Trypsin-EDTA and DMEM F12 mixture (1:3 ratio) treatment for 25 min (Lin et al., 2017). The cells were then collected and subjected to two-fold centrifugation at 500 *g* for 2 min and trituration in cell culture media. The resulting cell suspension was then passed through a syringe and needle, and plated in a 75 cm^2^ flask at a ratio of 1 plate: 1 flask. The flask cultures were then incubated for 1 week at 37°C and 5% CO_2_ prior to another round of isolation and re-seeding onto microelectrodes, with cell culture media changed twice in that week.

### 2.3 PDMS ring fabrication

**FIGURE 1:**
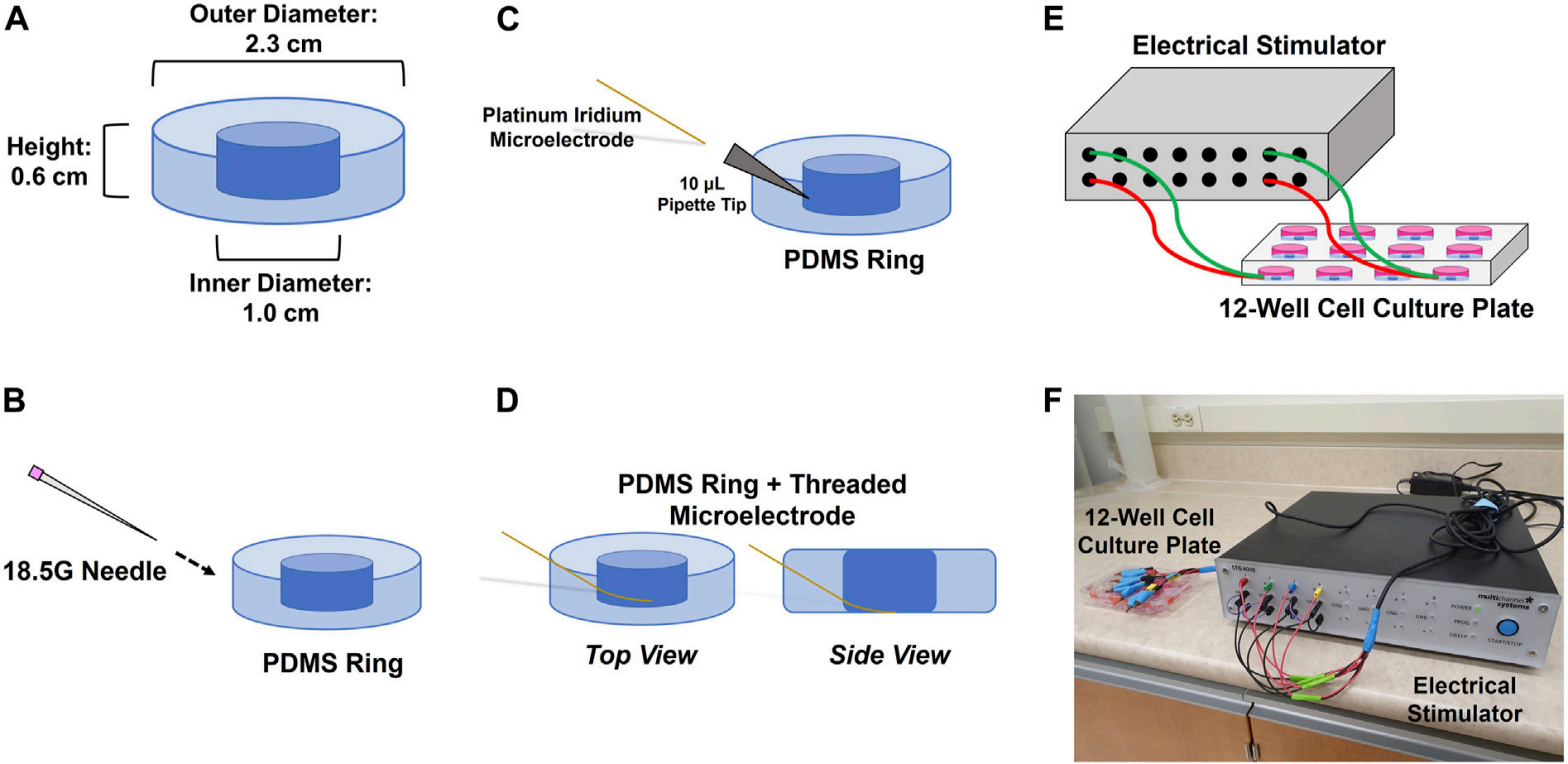
Procedure for inserting 75 μm microelectrodes into the sides of PDMS rings. **(A)** PDMS rings are cured to fit inside the well of a 12-well plate; **(B)** rings are punctured from the side with an 18.5 G needle at a 45° angle; **(C)** 10 μL pipette tip is fitted inside the punctured hole and the electrode is threaded through the pipette tip; **(D)** the electrode is threaded through to the point where its deinsulated tip is able to make contact with the bottom of the well once the ring is inserted. The pipette tip is removed once the electrode is threaded through. Each threaded electrode/PDMS ring assembly is then inserted into a 12-well culture plate that is connected to an electrical stimulator using clamped cables **(E,F)**.

To stabilize electrode placements in the 12-well plates, custom polydimethyl siloxane (PDMS) rings were created to prevent movement of the wires within the wells (Figure 1). PDMS elastomer base and curing agent were mixed together in a 50 mL tube in a 10:1 ratio, and left to set in the wells of a 12-well plate (2 g/well). Following curing for 2.5 h in an oven at 70°C, the resulting PDMS discs were extracted from the wells, hole-punched, and placed in a large 3 L beaker (50% methanol/50% water) under a fume hood overnight to wash out any toxic byproducts resulting from the curing process. Following this, the rings were submerged in water and autoclaved in preparation for use in cell culture.

### 2.4 Microelectrode fabrication

Platinum-iridium microwires (75 µm diameter) were used for fabrication of microelectrodes. Briefly, microwires were cut ∼15 cm in length. The insulation layer of the microwire tips was removed using nanosecond laser pulses (wavelength = 248 nm, energy = 150 mJ, beam attenuation = 5%, repetition rate = 10 Hz; COMPex 110, Coherent, CA, United States). The deinsulated region of the microwires was cut using a scalpel blade leaving 300–400 µm of bare metal at the tip. The tips of the microwires were then mechanically bevelled using a microelectrode beveler (BV-10, Sutter, CA, United States) to an angle of approximately 15°. Microelectrodes were then placed in 15 mL centrifuge tubes (Fisherbrand, Pittsburgh, PA, United States) filled with DI water and Alconox detergent, and treated in an ultrasonic cleaner for 30 min to remove the metal debris formed during the mechanical polishing step. The microelectrodes were then sonicated for another 30 min in DI water and rinsed with 70% ethanol. Stranded stainless steel wires were manually de-insulated to expose approximately 4-5 cm and were used as the counter electrodes.

### 2.5 Electrode plate setup

Insertion of microelectrodes into the PDMS rings and placement of the rings into the 12-well plates was all done within the aseptic environment of a biosafety cabinet. An 18.5G needle was used to puncture a hole through the side of a ring at a 45° angle. A 10 µL pipette tip was then fitted though the hole, and a microelectrode was threaded through the pipette tip such that the deinsulated end of the wire lay in the inner hole of the PDMS ring (Figure 1). The pipette tip was then withdrawn to effectively embed the insulated portion of the microelectrode in the side of the ring. The ring and microelectrode were then dipped in 70% ethanol, placed in one of the wells of a 12-well plate, and left to dry to form a sterile seal in the well. This also allowed the de-insulated tip of the wire to make contact with the bottom of the well. The insulated portions of the electrodes were then taped down over the edge of the 12-well plate to prevent further movement. Counter electrodes were placed on top of the PDMS rings and held down over the edge of the plate with tape on the day of the experiment.

Cells were isolated from the flask as above using diluted Trypsin-EDTA/DMEM F12, seeded at a density of 70,000 cells/well, and left to settle and incubate for 7 days at 37°C and 5% CO_2_ prior to the start of electrical stimulation. Cell culture media (DMEM/10% FBS/1% PS) was changed twice during the 7-day incubation period.

### 2.6 Electrical stimulation experiments

The cells were electrically stimulated for a 4 h duration each day over a total of 3 days using a paradigm adapted from *in vivo* ISMS work (Bamford et al., 2010). An STG4008 electrical stimulator (Multi Channel Systems MCS GmbH, Reutlingen, Germany) was used to electrically stimulate the cells, with programming of the stimulation patterns done through the MC_Stimulus II software. Cells were stimulated using a biphasic charge-balanced cathodic-first rectangular waveform, at an amplitude of 0 mA, 0.15 mA or 1.5 mA, 200 µs pulse duration, and 25 Hz. The charge injected per phase at 0 mA, 0.15 mA, and 1.5 mA was 0 nC, 30 nC, and 300 nC, respectively.

### 2.7 Immunofluorescence microscopy

Following 3 days of stimulation, glial cells were fixed with 5% formalin at 37°C for 10 min and washed three times with PBS. Cells were permeabilized with 0.1% Triton X-100 (TX100) in PBS and 10% Equine Serum (ES) for 2 h. Following this, the cells were incubated overnight at 4°C with rabbit anti-IL-1β (1:1000) and chicken anti-GFAP (1:5000) primary antibodies plus 1% ES. The cells were then washed three times with PBS, and incubated for 2 h at room temperature with goat anti-chicken Alexa Fluor 546 (1:200) and donkey anti-rabbit Alexa Fluor 647 (1:200) secondary antibodies plus Hoescht 33342 (1:1000) and 1% ES. The cells were then washed three times with PBS. Fluorescence microscopy was carried out on a Leica TCS SPE confocal microscope (Wetzlar, Germany). Components labelled included Hoescht for cell nuclei, enhanced green fluorescent protein (EGFP) expressed from transgenic microglia, glial fibrillary acidic protein (GFAP) for astrocytes, and interleukin-1 beta (IL-1β) as a pro-inflammatory biomarker. Analysis of fluorescence microscopy images was carried out with ImageJ (National Institutes of Health, Bethesda, MD, United States) using a custom macro measuring for fluorescence intensity and area coverage of biomarkers. Area coverage measured the total geometric surface generated by each biomarker from the cells in the image’s field of view. Fluorescence intensity was calculated by dividing the image-wide sum of each pixel intensity value for a biomarker divided by the area coverage of that biomarker in that image. Cell density was calculated by counting the number of nuclei found in each image. These metrics (fluorescence intensity, area coverage, cell density) were expressed as fold change against control wells with no wire. Zonal analysis (i.e., how outputs change as a function of distance from the electrode tip) was carried out at prescribed circular radii from the electrode tip (r = 50 μm, 100 μm, and 250 μm) (Mohammed et al., 2020). This was compared to data analyzed from the full frame of the image (734.05 μm × 734.05 μm).

### 2.8 Scanning electron microscopy

Qualitative assessment of damage to electrodes was carried out using a ThermoFisher Phenom XL Desktop scanning electron microscope (SEM) (Waltham, MA, United States). Images were acquired using backscattered electron detection at 610x and 4000x magnification. Elemental makeup of the electrode surfaces was quantified using the energy-dispersive x-ray spectroscopy (EDS) add-on to the SEM, at 4000x magnification.

### 2.9 Statistical analyses

All experiments were analyzed with a sample size of six (*n* = 6) with 2 internal replicates for each independent experiment. For statistical analysis of fluorescence intensity and area coverage of biomarkers and cell density, a two-way analysis of variance (two-way ANOVA) with Bonferroni’s *post hoc* test was performed. The independent variables analyzed were electrical stimulation amplitude and distance away from the tip of the electrode. For statistical analysis of EDS data, a one-way analysis of variance (one-way ANOVA) with Tukey’s *post hoc* test was performed using GraphPad Prism 10 (San Diego, CA, United States) with electrical stimulation amplitude being the main effect analyzed.

## 3 Results

### 3.1 Cellular responses at electrode interface

**FIGURE 2:**
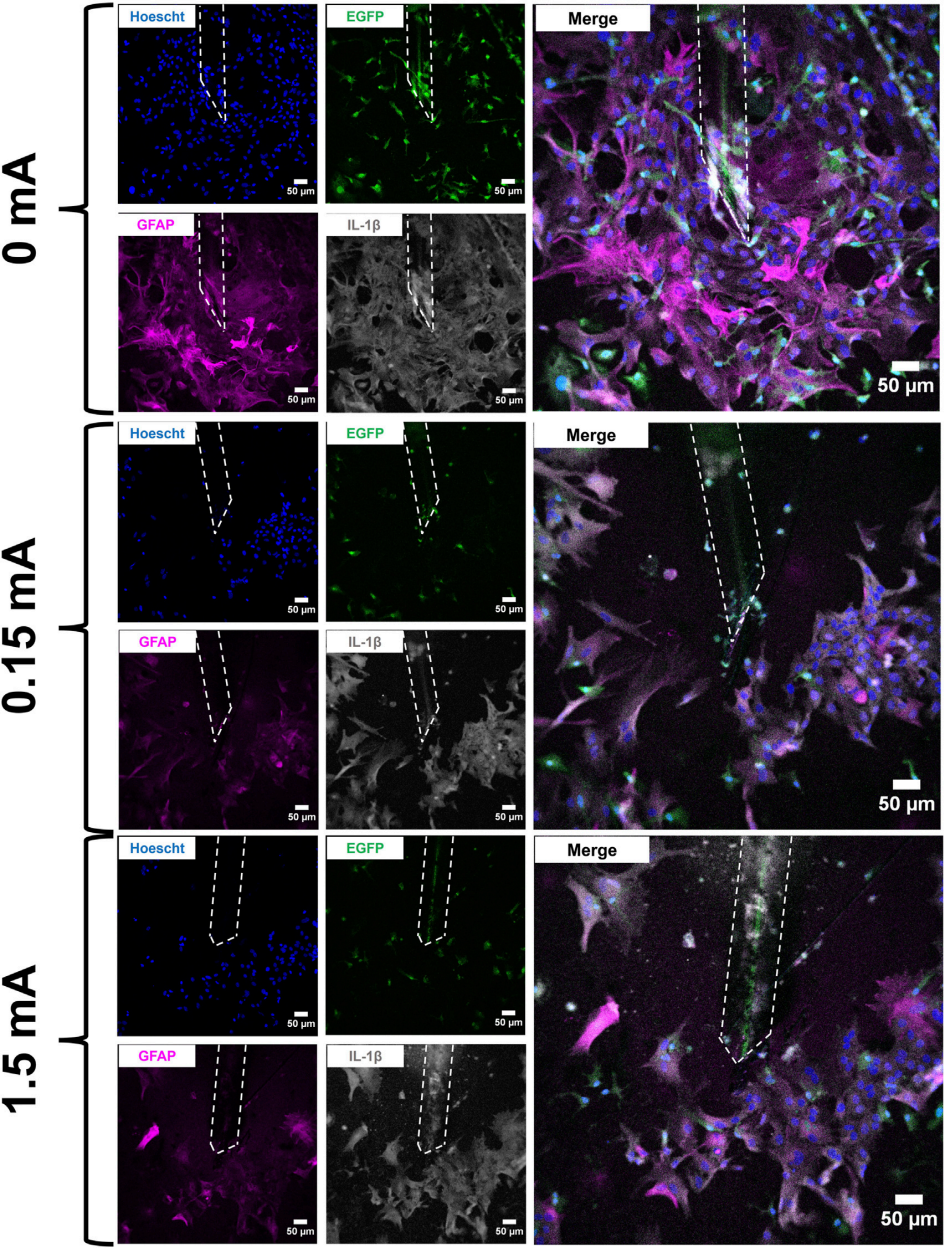
Immunofluorescent images of mixed glial cell cultures at the electrode interface following stimulation experiments (4 h/day × 3 days). Electrodes are marked by the white dashed outline in each image. Cell cultures were labelled with Hoescht 33342 (blue), EGFP (green), GFAP (magenta), and IL-1β (grey). An enlarged merged channel overlay of each condition is shown on the far right. Scale bars: 50 µm.

Mixed glial cell cultures were electrically stimulated using a biphasic charge-balanced rectangular waveform paradigm for 4 h daily over a short timecourse of 3 days. The cells were then fixed, immunolabelled and imaged on a confocal fluorescence microscope. Images were acquired across all stimulation amplitudes tested (0 mA, 0.15 mA, and 1.5 mA) (Figure 2).

**FIGURE 3:**
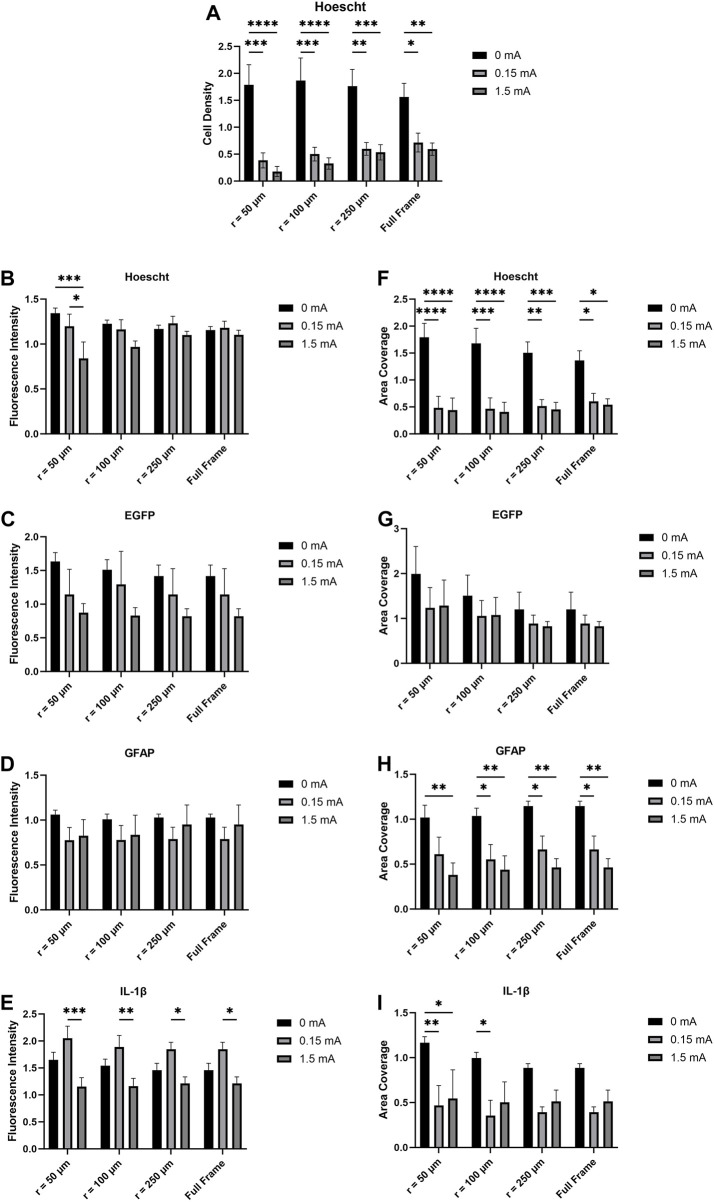
**(A)** Cell density, **(B,C,D,E)** fluorescence intensity, and **(F,G,H,I)** area coverage profiles of immunofluorescent images as a function of electrical stimulation current and distance from the electrode tip. Values are expressed as fold change *versus* no-wire control cell cultures (*n* = 6, two-way ANOVA with Bonferroni *post hoc* test). *p < 0.05, **p < 0.01, ***p < 0.001, ****p < 0.0001; Data = means ± SEM.

For fluorescence intensity and area coverage (Figure 3), significant main effects were detected across all biomarkers with the exception of GFAP fluorescence intensity and EGFP area coverage. *Post hoc* analyses revealed significant differences between different distances from the electrode tip and stimulation amplitudes in Hoescht fluorescence intensity, IL-1β fluorescence intensity, Hoescht area coverage, Hoescht cell density, GFAP area coverage, and IL-1β area coverage.

### 3.2 Electrical stimulation-induced electrode damage

**FIGURE 4:**
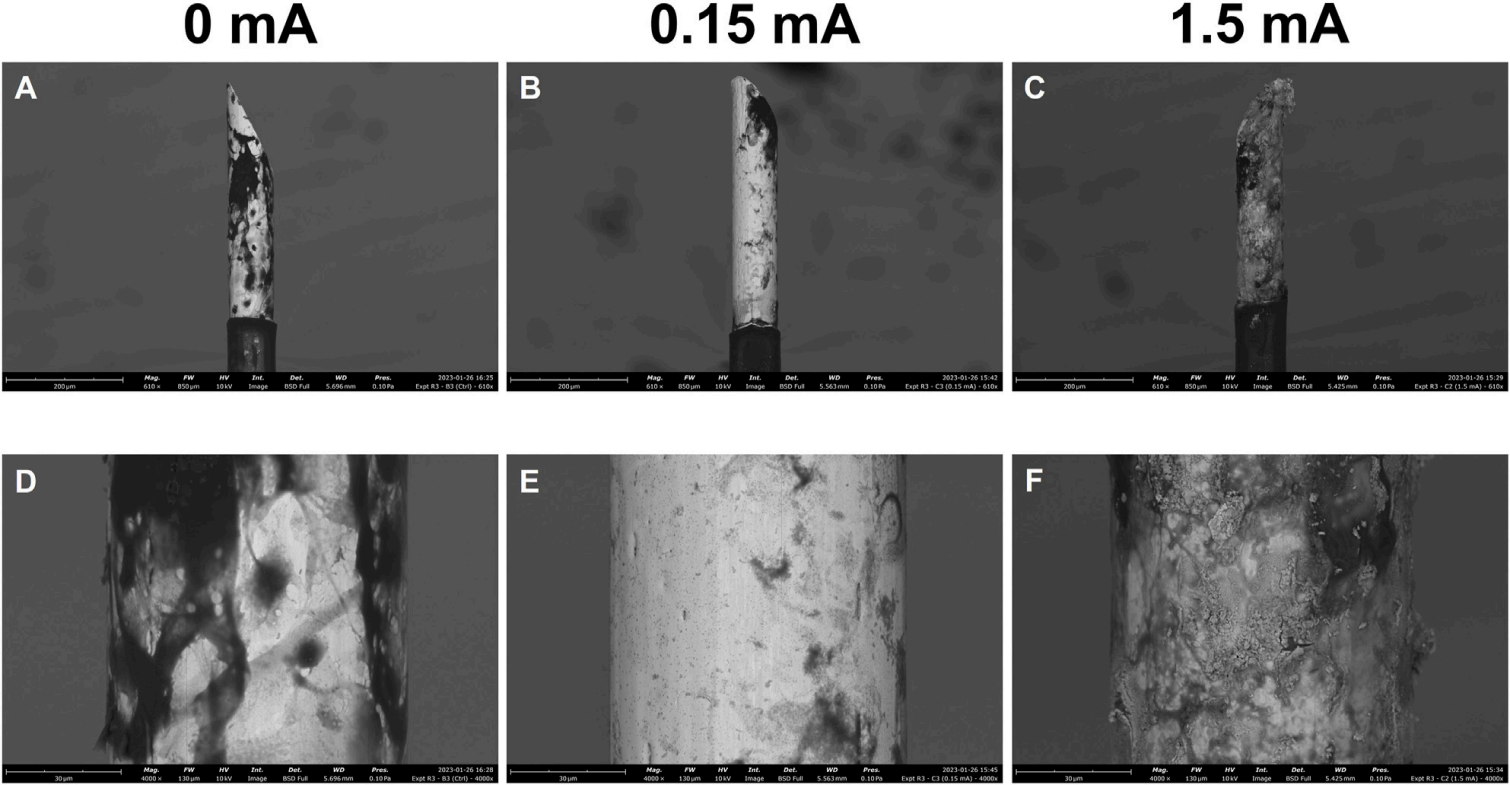
Scanning electron micrographs of electrodes following 3-day stimulation experiments. Stimulation amplitudes through the electrodes were 0 mA, 0.15 mA, and 1.5 mA. Images were acquired at 610x **(A,B,C)** and 4000x **(D,E,F)** magnification. Scale bars: 200 µm (610x), 30 µm (4,000x).

Following immunolabelling and confocal fluorescence microscopy, the electrodes were extracted from the cell culture wells and imaged on an SEM to qualitatively assess damage caused by the stimulation experiments (Figure 4). The SEM images for the 0 mA electrodes (Figures 4A, D) are best described as having large amounts of non-conductive deposits on their surfaces. In the 0.15 mA electrodes (Figures 4B, E), lesser amounts of such deposits were seen, but the overall shape of the electrode was intact. At 1.5 mA, however, deformation of the entire de-insulated tip of the electrode was apparent with the surface appearing warped and crateriform (Figures 4C, F).

**FIGURE 5:**
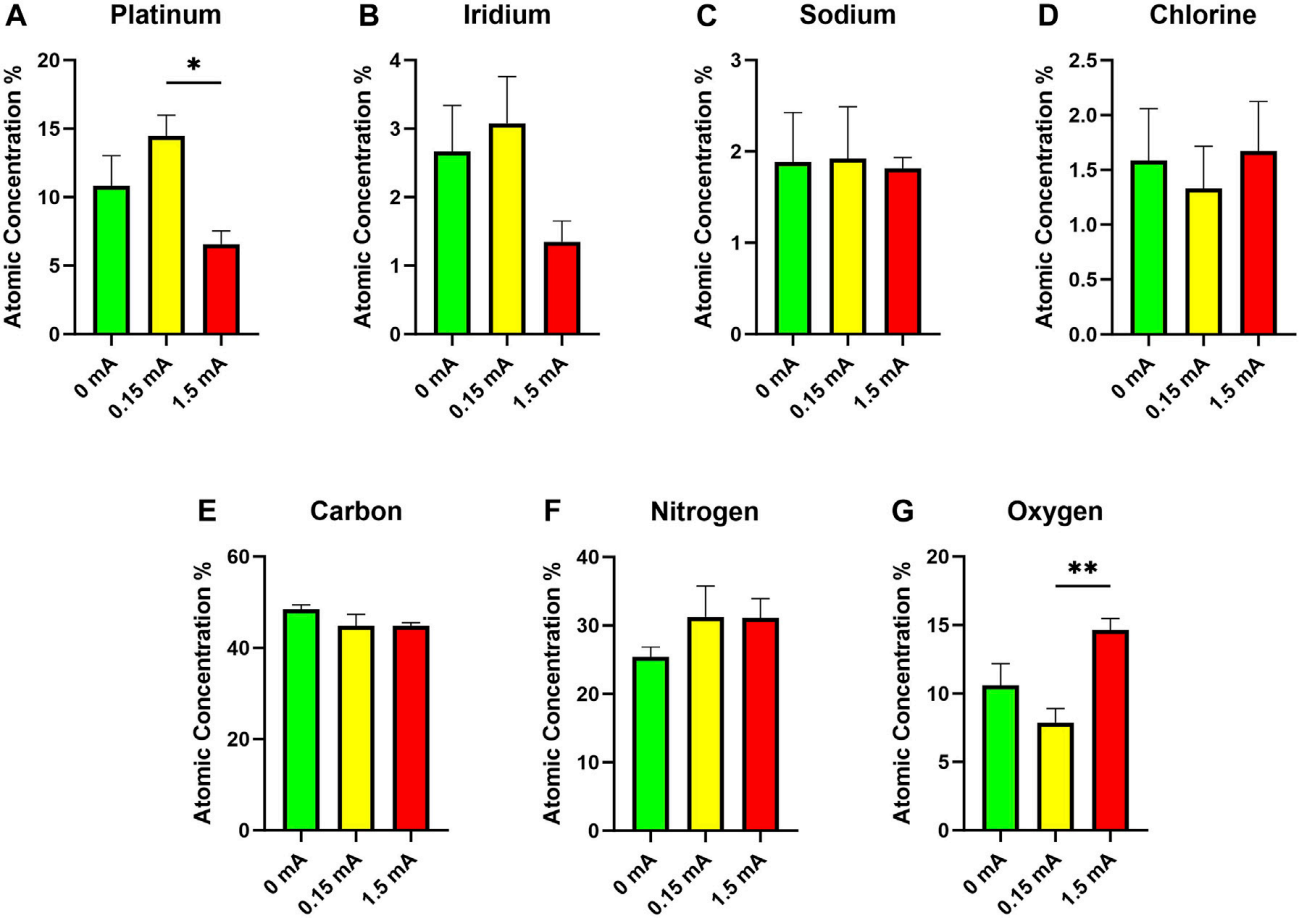
Energy-dispersive x-ray spectroscopy data showing atomic concentrations of **(A)** platinum, **(B)** iridium, **(C)** sodium, **(D)** chlorine, **(E)** carbon, **(F)** nitrogen, and **(G)** oxygen on the surfaces of electrodes following 3-day stimulation experiments (n = 6, one-way ANOVA with Tukey’s *post hoc* test). *p < 0.05, **p < 0.01; Data = means ± SEM.

In addition to SEM, EDS was used to quantify the elemental composition of the surfaces of the electrodes across the different experimental conditions (Figure 5). The most notable differences in composition were between the 0.15 mA and 1.5 mA conditions. Electrodes subjected to 1.5 mA of current had a much lower proportion of platinum at their surfaces compared to at 0.15 mA (Figure 5A). No significant differences were found across stimulation conditions for iridium (Figure 5B). Trace amounts of elemental sodium and chlorine (Figures 5C, D) are likely from the use of PBS, an aqueous salt solution, while the fixed cell cultures were in storage. Atomic concentrations of carbon across all conditions were similar (Figure 5E). No significant main effects were detected in the nitrogen group across the different stimulation conditions (Figure 5F). There was a statistically significant finding with oxygen (Figure 5G), with higher concentrations detected in the 1.5 mA condition *versus* the 0.15 mA condition.

## 4 Discussion

The goal of this work was to examine the effects of electrical stimulation on mixed glial cell cultures at the interface of a microelectrode designed for invasive stimulation of central nervous tissue. The prime objective of our experiments was to recapitulate *in vitro* the foreign body response that is orchestrated by microglia and astrocytes—namely, the formation of a glial scar that has been observed *in vivo* (Bamford et al., 2010; Salatino et al., 2017; Gulino et al., 2019; Otte et al., 2022). Previous studies successfully used *in vitro* methods to demonstrate the glial cell response (Polikov et al., 2006). We present a refined system with the integration of electrical stimulation into the experimental model as well as a hybrid cell biology-engineering approach to assessing damage to both physiologically relevant primary cells and electrodes.

In our experiments, stimulation paradigms of differing currents (0.15 mA vs. 1.5 mA) were compared while keeping other factors, such as cell culture composition and microelectrode material and geometry, constant. In other words, the variable manipulated was the amount of electrical charge delivered through the electrode interface with every pulse. Biphasic electrical pulse paradigms are pervasive in invasive implants as they are designed to mitigate tissue damage by cycling electrical charge out of tissue through the use of a second phase of opposite polarity (Weitz et al., 2014). The low current of 0.15 mA was selected for this experiment as it is considered a safe, physiologically relevant current in *in vivo* experiments; it has been shown in previously published ISMS work, for example, that currents near or at this magnitude passed by microelectrodes of similar design to the ones in our study are capable of activating interneurons at the lumbar enlargement and eliciting load-bearing movements or other effective functions in animals (Bamford and Mushahwar, 2011; Holinski et al., 2016; Mercier et al., 2017; Dalrymple et al., 2018; 2020). In contrast, a current of 1.5 mA would not be appropriate for *in vivo* work using such microelectrodes. That amount of current would risk not only excessive activation of the stimulated limbs, but also substantial damage to the stimulated tissue. The 1.5 mA amplitude was selected for this study as a means of inducing maximum damage to the cell cultures (i.e., a worst-case scenario).

Although the stimulation paradigms used in our experiments are adapted from previous *in vivo* ISMS work, the experimental platform and workflow presented have a wider reach. They can accommodate testing of additional stimulation parameters (e.g., pulse width, frequency, waveform shape) as well as different electrode materials, sizes, and geometries. Although such parameters can also be tested *in vivo*, an *in vitro* approach allows us to conduct these experiments with relatively higher throughput and with a reduced ethical footprint. Invasive electrical stimulation of nervous tissue has applications in treating a large breadth of diseases and injuries (Hachmann et al., 2013; Miocinovic et al., 2013; Dolbow et al., 2021); assessing electrical stimulation-induced damage on glial cells and finding ways in which to modulate glial cell response by modifying both implant and stimulation paradigm designs are thus valuable research goals for stimulation targets in the brain or spinal cord.

### 4.1 Fluorescence imaging analysis

Fluorescence intensity, a measure of the image-wide sum of the pixel intensity values for a biomarker divided by the area coverage of that biomarker, was calculated for each immunofluorescence image. Significant differences with Hoescht were detected at 50 μm from the electrode thus suggesting that electrical stimulation does have a localized effect on Hoescht expression in mixed glia (Figure 3B). No significant differences were detected in the EGFP and GFAP fluorescence intensity data as a function of either stimulation current or distance from the electrode (Figures 3C, D). However, the IL-1β fluorescence intensity data (Figure 3E) suggest that not only does electrical stimulation significantly downregulate IL-1β production but that also this observation is detectable even when measuring the full image as opposed to a subset of it. *In vivo* findings revealed that electrical stimulation did not lead to additional upregulation in inflammatory biomarkers in electrically stimulated animals compared to unstimulated animals (Bamford et al., 2010). However, it is worth noting that the levels of IL-1β seen in 0 mA, 0.15 mA, and 1.5 mA seen are all above that of a control that we used where there was no wire. This suggests that the mere presence of the microelectrode in the cells is capable of eliciting upregulation of inflammatory biomarkers such as IL-1β (Srinivasan et al., 2004; Green and Nolan, 2012; Iravani et al., 2012; Minogue et al., 2012), but that additional stimulus in the form of electrical current does not induce further significant biomarker upregulation even at the interface. Such observations were also made in Bamford et al.’s study (Bamford et al., 2010). In that study, the encapsulation responses by reactive GFAP-positive astrocytes as well as inflammatory responses were attributed in large part to the insertion of the wires and not to subsequent repeated electrical stimulation. The current study draws inspiration from the stimulation paradigm and microelectrodes used in Bamford et al.’s study. IL-1β is a documented pro-inflammatory biomarker associated with neuroinflammation and is expressed by both microglia and astrocytes. Our study revealed differences in IL-1β fluorescence intensity as a function of stimulation current up to the full size of the images measured—this supports evidence suggesting that IL-1β is upregulated in astrocytes and microglia in a wide range of diseases and injuries (Shaftel et al., 2008; Liu and Quan, 2018; Mendiola and Cardona, 2018). The highest IL-1β signals were seen in the 0.15 mA condition compared to the 1.5 mA condition—peri-electrode void formation at 1.5 mA likely explains the lower IL-1β signal at that current. GFAP is a biomarker associated with the cytoskeleton of astrocytes; its upregulation is associated with astrocyte response to injury or disease (Pekny and Nilsson, 2005; Brahmachari et al., 2006; Ben Haim et al., 2015; Rosskothen-Kuhl et al., 2018). However, in the current study, analyses of GFAP fluorescence intensity showed no significant differences between the different stimulation conditions and across the different distances from the electrode tip.

Immunofluorescence images were also acquired and measured for area coverage—the total geometric size of a biomarker’s signal across all cells present in the image’s field of view. From the images acquired of the cell cultures interacting with the electrodes (Figure 2), a large mass of both microglia and astrocytes were observed at and around the electrodes. More specifically, microglia in the 0 mA images have been seen in very close proximity to the surface of the electrode while larger concentrations of astrocytes were seen further afield from the electrode. This is in line with established knowledge on how glial scar formation occurs; specifically, microglia are known to migrate to a lesion or foreign body first and facilitate migration of astrocytes to the site of interest (Carbonell et al., 2005; Gao et al., 2013; Lind et al., 2013; Donat et al., 2017; Shinozaki et al., 2017; Zhou et al., 2023). The two cell types then work together to cordon off the site from any nearby healthy tissue. *In vivo*, glial scarring takes place over the course of several weeks (Fitch et al., 1999; Tran et al., 2022). Given our experimental design and timecourse, we are only able to model an early response that is consistent with the glial scarring process and not the glial scar itself. When electrical stimulation is used, however, our image data suggest a localized disruption of the aggregation of cell bodies around the electrode. The Hoescht area coverage data suggest that electrical stimulation, even at a low current of 0.15 mA, significantly reduces mixed glia area coverage compared to the 0 mA condition even when the entirety of each image (734.05 μm × 734.05 μm) was measured (Figure 3F). The trend observed from this holds true even when calculating cell density from the Hoescht image data (Figure 3A). Although no differences were detected in the EGFP area coverage data with respect to stimulation conditions or distance from electrode (Figure 3G), GFAP area coverage data suggests reduced area coverage with 0.15 mA and 1.5 mA conditions vs. 0 mA even when the full frame of the image was measured (Figure 3H). On the other hand, the IL-1β area coverage data (Figure 3I) suggests a more localized effect, with significant differences between stimulation conditions detected up to a distance of 100 μm from the electrode tip. The gap devoid of cells (plus evidence of autofluorescent debris) was expected for the 1.5 mA stimulation amplitude as it was an extremely high current for the size of electrode that was used; however, it was not expected that this gap would also be present in the 0.15 mA case as it is a safer and more physiologically relevant stimulation amplitude that was tolerated *in vivo* (Bamford et al., 2010; Mercier et al., 2017). The data suggest a trendwise decrease in area coverage with the 0.15 mA and 1.5 mA stimulation conditions compared to 0 mA—we believe the formation of the voids around the electrodes as a result of stimulation is reflected in this drop in area coverage. The observation that electrical stimulation reduces cell coverage at the interface presents two possible scenarios as to the fate of the cells that otherwise would have been at the interface: 1) the cells at the interface had died as a result of the electrical stimulation, or 2) the cells at the interface had migrated away from the interface as a result of the stimulation. While the methods described in this study do not allow us to determine if one or the other scenario occurred, follow-up live cell imaging experiments that take advantage of transgenic EGFP expression in the microglia will enable us to track cell movements and behaviour over a 4-h stimulation time course at the electrode interface.

### 4.2 SEM analysis

In addition to damage to the glial cells at the interface, damage to the electrodes themselves as a result of stimulation was assessed. SEM enables close inspection of the microstructure of a surface material. The non-conductive deposits seen in abundance on the 0 mA electrodes (Figures 4A, D) are likely residual organic matter (i.e., cells) that were attached to the surfaces of these electrodes. The images for this condition support the findings from the immunofluorescence images of the 0 mA condition, and reinforce the idea that the glial cells congregate at and around the electrode as part of a foreign body response (Polikov et al., 2006; Carnicer-Lombarte et al., 2021; Sharon et al., 2021). Electrical stimulation at 0.15 mA (Figures 4B, E) resulted in less of these organic deposits covering the surface of the electrode, but the current was otherwise not intense enough to cause deformation and warping of the material at the surface. Applying an extreme current of 1.5 mA, on the other hand, caused visible deformation and corrosion of the electrodes (Figures 4C, F). With this comes a change in the surface material composition and geometric surface area of the electrodes (Brummer and Turner, 1977; Merrill et al., 2005; Cogan, 2008; Green et al., 2014; Cui et al., 2019; Shepherd et al., 2021)—such a change at just 3 days of usage would of course suggest that 1.5 mA is an inappropriate level of current to be passed through these microelectrodes.

### 4.3 EDS analysis

The EDS, which measures elemental composition of the surface of the material, also provides information regarding the types of deposits on the surface of the electrode as well as potential reaction byproducts arising from electrochemical reactions. A higher proportion of oxygen (and lower proportions of platinum) seen for the 1.5 mA electrodes was likely due to an increase in irreversible oxidation induced by such a high current (Figures 5A, G) (Merrill et al., 2005). No differences in levels of these elements were detected between the 0 mA and 0.15 mA conditions, thus suggesting that any redox reactions that occurred at the interface were reversible and did not result in permanent oxidation of the electrodes. A higher proportion of carbon was expected from the 0 mA electrodes compared to the other conditions as suggested by the non-conductive organic deposits seen from the SEM images. Cells and extracellular matrices are primarily comprised of carbon compounds hence our hypothesis that the 0 mA electrodes would have a higher carbon content (Venugopal et al., 2008; Akinnuoye et al., 2011). The high atomic carbon readings across all conditions tested (Figure 5E) were thus contrary to what we had expected. However, the uniform atomic concentration of carbon across all electrodes examined was possibly the result of the cell cultures, electrodes included, being subjected to the same types and concentrations of organic compounds in the fixation and immunolabelling processes (e.g., formalin, ES, antibodies)—to preserve the integrity of the cell cultures following experiments, our electrodes were extracted only after the immunolabelling and fluorescence microscopy steps.

### 4.4 Charge injection as a damage mechanism

Also of interest to us are the potential mechanisms of damage inflicted upon cells as a result of electrical stimulation. Irreversible redox reactions, as previously mentioned, could potentially lead to leaching of cytotoxic byproducts into the cell culture media and have an adverse effect on nearby glial cells (Merrill et al., 2005). The amount of electrical charge delivered in a pulse is a function of stimulation amplitude and pulse width, and would also have an effect on cellular response. The charge injection capacity for Pt-Ir electrodes has previously been quoted to be in the range of 50–150 μC cm^−2^·ph^−1^ (Rose and Robblee, 1990). Factoring in the geometric surface area of the deinsulated portion of our Pt-Ir microelectrodes (approximately 89,000 μm^2^) suggests that supplying a current of 1.5 mA will far exceed this charge injection capacity range, while a current of 0.15 mA (approximately 33.7 μC cm^−2^·ph^−1^) will not. Charge density is an important parameter to calculate when designing neural electrodes as the size and material of electrodes affect design safety limits (McCreery et al., 1990; Merrill et al., 2005; Fairfield, 2018). Other previous reports document using stimulating electrodes at varying charge densities across different materials (e.g., platinum, iridium oxide, platinum-iridium, stainless steel, PEDOT/polypyrrole nanotubes) (Harnack et al., 2004; Abidian et al., 2010; Harris et al., 2017; 2018; Sikder et al., 2021)—comparisons made in these works highlight the significance of material selection in electrode design as well as the differences seen in terms of charge injection, development of toxic byproducts, and tissue damage. In McCreery et al.’s work, circular platinum disk electrodes of varying sizes (0.01–0.1 cm^2^) were subjected to charge injection of 1 μC (i.e., charge densities ranging between 10–100 μC cm^−2^·ph^−1^) with the goal of confirming and studying the effects of electrode size and charge density on neuronal injury (McCreery et al., 1990). In the same study, penetrating microelectrodes (6.5 ± 3 × 10^5^ cm^2^) were injected with current resulting in a geometric charge density of 800 μC cm^−2^·ph^−1^—although this was beyond the referenced safe range quoted in Rose and Robblee, it was also mentioned in the study that the microelectrodes were subjected to potentiodynamic cycling to increase charge capacity and reduce electrode dissolution (McCreery et al., 1990). Charge injection into tissue, or in this case cell cultures, is intended to elicit action potentials in neurons. In the case of glia, it is possible that even biphasic charge injection can cause charge imbalances along the membranes of cells. This in turn may trigger activation of glial ion channels, voltage-gated or otherwise, in an effort to restore perturbed membrane potentials back to their resting values (Nowak et al., 1987; Verkhratsky and Steinhäuser, 2000). Doing so may also cause cells to become damaged through changes in tonicity (Turner and Sontheimer, 2014). Characterizing the microelectrode’s capabilities with regards to impedance, charge storage capacity, and charge injection limit are important to better understanding some of the mechanisms behind the sorts of electrochemical phenomena that take place at the electrode-cell culture interface as well as the limits to which the electrodes can be stimulated before corrosion occurs (Merrill et al., 2005; Cogan, 2008).

### 4.5 Limitations of study

The current study uses primary mixed glial cell cultures as a way of exclusively studying glial cell response to electrode presence and electrical stimulation. This reductionist approach to controlling for variability brought on by other factors also means that there is a limit to how much this *in vitro* model is representative of *in vivo* physiology. Although neurons were not included in the cell culture model, Bamford et al. did note that a local (but statistically insignificant) increase in NeuN in their electrically stimulated animals (Bamford et al., 2010). Other structures, such as the blood-brain barrier, were also not modelled in the current study. *In vitro* cell density is also less compared to *in vivo*. In our experimental design, routine refreshes of cell culture media and a finite space for cell cultures to grow in prevented us from examining longer term (e.g., 30 days) effects that could otherwise be done in animals or other cell culture models (Potter and DeMarse, 2001; Lichtenstein et al., 2018; Gilmour et al., 2019). A seeding density of 70,000 cells/well was selected to provide a confluent amount of cells in the centre of each cell culture well at the time of the start of the experiment. The space in each well that was available for cell culture (0.79 cm^2^) was much smaller than the total surface area of the well itself (4.15 cm^2^) due to the presence of the PDMS ring (Figure 1). In our design there was therefore limited space available for cell culture in each well thus limiting potential culture time prior to passaging or fixation. Furthermore, the electrodes were inserted into the plates first before cell seeding took place (i.e., a stab wound scenario was not captured in the experiments). When inserting the PDMS rings and electrodes into the 12-well plates, they were first sterilized in 70% ethanol as such work takes place in a biosafety cabinet. Previous trial work we conducted saw cell death from potential residual ethanol as well as the PDMS rings sitting on top of cells that would have already been seeded in the plate. For this reason we elected to embed the PDMS rings and electrodes in the plates first before cell seeding.

Despite the limitations of conducting *in vitro* studies as described above, the *in vitro* results reported herein agree well with results reported from previous *in vivo* studies - although inflammatory responses were observed in response to the presence of the electrodes themselves, applied electrical stimulation does not induce additional upregulation of biomarkers associated with a pro-inflammatory state. Specifically, in Bamford et al.’s study consistent applied electrical stimulation (48 nC/phase, 25 pulses per second) over a 30 days timespan induced no further damage in rat spinal cords than was found in unstimulated rats. This may also mean that astrocytes are more responsive to other cells’ reactions to electrical stimulation than react directly to the electrical stimulation itself (Linnerbauer et al., 2020; Han et al., 2021).

## 5 Conclusion

The experiments in the present work investigated the effects of electrical stimulation on mixed glial cell populations at the interface of Pt-Ir microelectrodes. An *in vitro* setup was used to evaluate the responses of primary mouse glial cells in a high-throughput setup that is designed to inform the design of *in vivo* experiments. The cellular responses to both the presence of the electrodes as well as applied electrical stimulation were captured.

The data presented herein suggest a large aggregation of microglia and astrocytes at the electrode interface, which is reminiscent of a foreign body response observed *in vivo*. When electrical stimulation is factored in, a lower density of cells at and around the electrode interface was observed. A previous *in vivo* study reported comparable findings in rats - although inflammatory biomarkers were upregulated in electrically stimulated animals, this was attributed to the initial insertion of the electrodes into tissue and cell reactivity to the electrode itself as opposed to reactivity from consistently applied stimulation over a maximum of 30 days (Bamford et al., 2010). Analysis of the fluorescence image data collected revealed differences in biomarker fluorescence intensity and area coverage as a function of both stimulation current intensity as well as distance from the electrode tip—taken together, this suggests that electrical stimulation of mixed glia induces localized responses around the electrode tip to varying degrees. *In vitro*, the microglia and astrocytes may be dying as a result of electrical stimulation or retreating away from the vicinity of the electrode. Live cell imaging using transgenic cells expressing EGFP can determine the fate of glial cells at the electrode interface as a result of different electrical stimulation paradigms in future experiments.

Varying stimulation parameters such as current, pulse width, frequency, and waveform pattern (e.g., rectangular, sinusoidal, ramped) can be readily investigated using the experimental approach developed in this work. Furthermore, additional data analysis such as microglia-astrocyte ratio changes, up- or down-regulation of other cytokines, etc. would greatly benefit from these proposed experiments examining the impact of various parameters. The types of electrochemical reactions at the interface as a result of varying stimulation parameters and factors such as electrode material composition, electrode geometry, and cell culture media, can also be determined.

The results generated from this work are intended to better inform device developers of neural interfaces of the biocompatibility and safety of invasive neural implants, allowing these devices to last longer and function more effectively in persons experiencing neurological disease or injury.

## Data Availability

The raw data supporting the conclusion of this article will be made available by the authors, without undue reservation.
